# Comparison of recruitment yield in prevention and therapy trials

**DOI:** 10.1186/1745-6215-14-S1-O112

**Published:** 2013-11-29

**Authors:** Cindy Cooper, Adjoa Asante, Danny Hind, Stephen Walyers

**Affiliations:** 1 University of Sheffield, Sheffield, UK

## Introduction

The literature demonstrates that only 31% of trials are successful in recruiting 100% of their original target and 45% recruit fewer than 80% (McDonald, 2006). It has been proposed that in prevention trials only one in twenty of patients screened are enrolled into trials and for medical care therapy trials the figure is nearer 1 in 5 (Spilker and Cramer, 1992).

## Aims

To compare recruitment in therapeutic trials with that in prevention trials to test Spilker and Cramer’s hypothesis that there is a large difference between the two types of studies.

## Methods

Data from all stages of the recruitment process were extracted from existing systematic reviews of prevention and therapy RCTs focussing on the use of metformin monotherapy in diabetes as a case study.

## Results

Twenty six diabetes prevention and twenty- seven diabetes therapy papers were reviewed. The recruitment yield, defined as the percentage of people recruited to people screened, in the diabetes prevention studies was 11%. The recruitment yield of the diabetes therapy studies was almost five times higher at 53%.

## Conclusions

Recruitment yields in prevention and therapy trials, were higher than Spilker and Cramer’s predictions of 5% and 20% respectively. However, there is a large difference between recruitment yields to the two types of studies and this should be taken into consideration when estimating recruitment rates for future trials. Reporting of data at all stages in the recruitment process needs to be improved to facilitate better estimation of recruitment yields.

